# Increased DNA microarray hybridization specificity using sscDNA targets

**DOI:** 10.1186/1471-2164-6-57

**Published:** 2005-04-22

**Authors:** Christopher S Barker, Chandi Griffin, Gregory M Dolganov, Kristina Hanspers, Jean Yee Hwa Yang, David J Erle

**Affiliations:** 1 Gladstone Institute of Cardiovascular Disease, The J. David Gladstone Institutes, San Francisco, California 94158, USA; 2 San Francisco General Hospital General Clinical Research Center, University of California, San Francisco, San Francisco, California 94143, USA; 3 Department of Medicine, University of California, San Francisco, San Francisco California 94143, USA

## Abstract

**Background:**

The most widely used amplification method for microarray analysis of gene expression uses T7 RNA polymerase-driven *in vitro *transcription (IVT) to produce complementary RNA (cRNA) that can be hybridized to arrays. However, multiple rounds of amplification are required when assaying very small amounts of starting RNA. Moreover, certain cRNA-DNA mismatches are more stable than the analogous cDNA-DNA mismatches and this might increase non-specific hybridization. We sought to determine whether a recently developed linear isothermal amplification method (ribo-SPIA) that produces single stranded cDNA would offer advantages over traditional IVT-based methods for microarray-based analyses of transcript expression.

**Results:**

A single round of ribo-SPIA amplification produced sufficient sscDNA for hybridizations when as little as 5 ng of starting total RNA was used. Comparisons of probe set signal intensities obtained from replicate amplifications showed consistently high correlations (r = 0.99). We compared gene expression in two different human RNA samples using ribo-SPIA. Compared with one round IVT, ribo-SPIA had a larger dynamic range and correlated better with quantitative PCR results even though we used 1000-fold less starting RNA. The improved dynamic range was associated with decreases in hybridization to mismatch control probes.

**Conclusion:**

The use of amplified sscDNA may offer substantial advantages over IVT-based amplification methods, especially when very limited amounts of starting RNA are available. The use of sscDNA targets instead of cRNA targets appears to improve hybridization specificity.

## Background

DNA microarrays are a powerful tool for global analysis of gene transcript expression. The initial studies using arrays required large amounts of starting material in order to reliably detect sample signals. Since that time, improvements in sample preparation, amplification and labeling methods [1-5] have reduced the starting material requirement to ~1–5 μg of total RNA [6]. Efforts to use smaller amounts of starting material have focused on PCR [7,8] and multiple rounds of T7 RNA polymerase *in vitro *transcription [IVT] [9-12]. PCR based methods have been successfully used to amplify subnanogram quantities of RNA from as little as a single cell [13,14], but these approaches have not been widely adopted. Most attempts to perform arrays using submicrogram amounts of RNA have relied on 2 or 3 rounds of linear amplification using IVT, but this approach has proven to be time consuming and technically demanding. In our hands, two round IVT is necessary to prepare samples from 5–50 ng total RNA and the amplification typically takes 4–5 days to complete. Others have reported a 10% decrease in sensitivity in detection of differentially expressed genes with the addition of a second IVT round [15].

**Figure 1:**
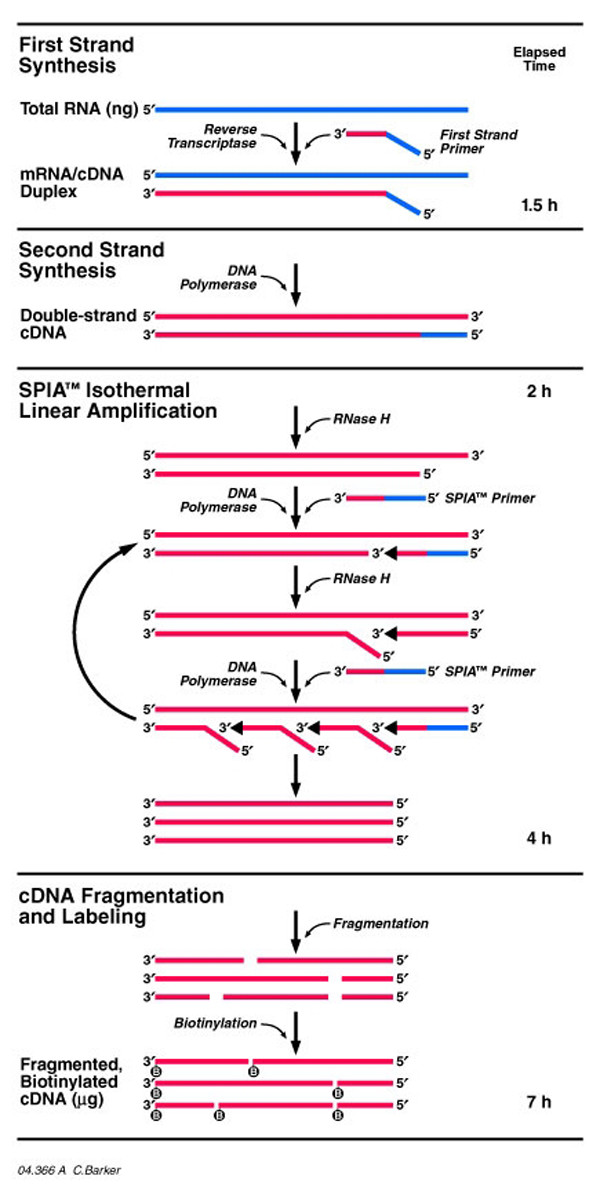
Diagram of the ribo-SPIA process for synthesis of sscDNA.

A new single primer, isothermal linear amplification method (ribo-SPIA) has been specifically developed for amplification of very small samples for use on DNA microarrays [16,17]. With this method (Figure 1), small amounts of total RNA are reverse transcribed into cDNA using a chimeric RNA/DNA primer containing oligo(dT) and a unique RNA sequence tag at the 5' end. Linear amplification requires the addition of RNase H, DNA polymerase and excess chimeric primer. The RNase H digests RNA from RNA/DNA hybrids thus exposing a single stranded binding site where a new copy of the primer anneals and the DNA polymerase initiates synthesis of a fresh copy of cDNA, displacing the original antisense strand of the cDNA. A single isothermal linear amplification reaction rapidly generates sufficient single-stranded cDNA (sscDNA) for multiple hybridization reactions. sscDNA samples are fragmented to provide sscDNA fragments of ~50–200 bp, end labeled with biotin and used for microarray hybridization. Approximately 100,000-fold amplification is typical for a single amplification step. The ribo-SPIA method is potentially attractive because the amplification can be completed in a single day and there are no purification steps until after completion of amplification, thus reducing the risk of losing sample during handling. In this study, we have investigated the utility of ribo-SPIA generated sscDNA for DNA microarray analysis of small starting samples. We assessed yield and reproducibility of sscDNA, and compared sscDNA-based microarray results with microarray results obtained using IVT amplification and with results of quantitative PCR in order to assess if this method conferred new advantages for use with DNA microarrays.

## Results and discussion

### Yield of sscDNA

We used the ribo-SPIA method to amplify several different total RNA samples. RNA was obtained from a pool of human tissues (Clontech Universal Human Reference RNA, or cUHR, Experiment 1), mouse liver (Experiment 2), a second pool of human cells (Stratagene Universal Human Reference RNA, or sUHR, Experiment 3), and K562 human erythroleukemia cells (also Experiment 3). The amount of starting total RNA ranged from 5–100 ng and yields were in the range of ~6–12 μg of sscDNA (Table I). The somewhat lower yields seen in Experiment 1 are likely attributable to our unfamiliarity with the protocol, and yields improved in subsequent experiments. There was no clear relationship between the amount of starting RNA and the sscDNA yield. To determine how much sscDNA product was produced in the absence of any template, we performed two additional amplifications with no input RNA (Experiment 4). Some sscDNA was produced, although the amount was substantially less than that seen when input RNA was present (Table 1). To determine the possible impact of this template-independent product on microarray results, we hybridized the entire sscDNA product from a template-independent reaction to a U95Av2 microarray. This resulted in low overall signal intensity with only 0.6% of probe sets yielding "present" calls.

**Table 1:** sscDNA yield from ribo-SPIA experiments

**Sample**	**Input RNA**	**sscDNA Total Yield (μg)**
Experiment 1		
cUHR 1	20 ng	5.7
cUHR 2	20 ng	7.3
cUHR 3	20 ng	4.6*
Experiment 2		
Mouse liver 1	5 ng	9.4
Mouse liver 2	5 ng	11.8
Mouse liver 3	5 ng	10.0
Mouse liver 4	100 ng	11.4
Mouse liver 5	100 ng	10.5
Mouse liver 6	100 ng	8.7
Experiment 3		
K562 1	10 ng	6.6
K562 2	10 ng	7.8
K562 3	10 ng	8.3
sUHR 1	10 ng	8.4
sUHR 2	10 ng	9.4
sUHR 3	10 ng	7.8
Experiment 4		
No Input RNA 1	0	2.7
No Input RNA 2	0	3.2

*Part of sample lost during handling and was removed from later calculations.

### Size of sscDNA products

sscDNA preparations were analyzed by electrophoresis using an Agilent 2100 BioAnalyzer. sscDNAs ranged widely in size and the median size was typically slightly greater than 1 kb (data not shown). sscDNAs were fragmented in preparation for hybridization resulting in fragments of ~50–200 bp. These results are similar to those previously obtained using this method [16,17].

### Reproducibility of microarray hybridization results

**Figure 2:**
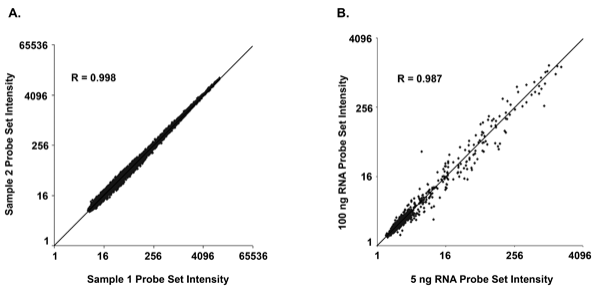
**Correlation of gene expression measurements for technical replicates prepared using the ribo-SPIA protocol. **(A) Data shown here are from two independent amplifications and hybridizations performed using the same starting RNA (cUHR, Experiment 1) and are representative of the pairwise correlations obtained for all replicate hybridizations in Experiments 1 – 3. (B) Data shown compare two independent amplifications using 20-fold different mass of starting RNA (5 ng versus 100 ng).

Each of the experiments included replicate amplifications (independent amplifications of aliquots of the same starting material). We hybridized each replicate to a separate microarray and calculated intensities for each probe set. Fig. 2A shows an example of one pair of replicate hybridizations from Experiment 1, each performed using 20 ng of cUHR RNA as starting material. Pairwise comparisons of probe set intensities for replicate hybridizations (Table 2) produced very high correlations (r = 0.983–0.996) across the entire range of starting RNA amounts used in the three experiments (5–100 ng). One previous study also showed high correlations (r ~ 0.97–99) between replicate array data produced using the ribo-SPIA method [16]. To assess how the amount of input RNA affects microarray results, we compared intensities found using 5 versus 100 ng of murine liver RNA (Experiment 2). When a 5 ng sample was compared to a 100 ng sample, there was a similar strong correlation (r = 0.987), indicating that large differences (20-fold) in starting material between reactions had small effects on measurements of gene expression (Fig. 2B). Previous reports have used correlation values between replicates to assess the reproducibility of other amplification methods. Those studies involve different laboratories and a wide range of microarray platforms, which makes direct comparison challenging. However, the correlations that we obtained using the ribo-SPIA method compare favorably with those reported for two rounds of IVT amplification (r = 0.92–0.98) [18-21], SMART amplification (r = 0.85–0.97) [22-24], and a PCR-based form of amplification (r = 0.97) [19].

**Table 2:** Correlations between signal intensities for replicate hybridizations

**Sample**	**Input RNA**	**Range of correlations**^1^
Experiment 1		
cUHR 1–2^2^	20 ng	0.996
Experiment 2		
Mouse liver 1–3	5 ng	0.983–0.993
Mouse liver 4–6	100 ng	0.986–0.991
Experiment 3		
K562 1–3	10 ng	0.985–0.990
sUHR 1–3	10 ng	0.983–0.991

^1^Correlation coefficients (r values) were calculated by examining all three possible pairwise comparisons between replicates.

^2^The third replicate from this set was removed from all calculations due to losses of material during sample preparation.

### Differential gene expression measurement

**Figure 3:**
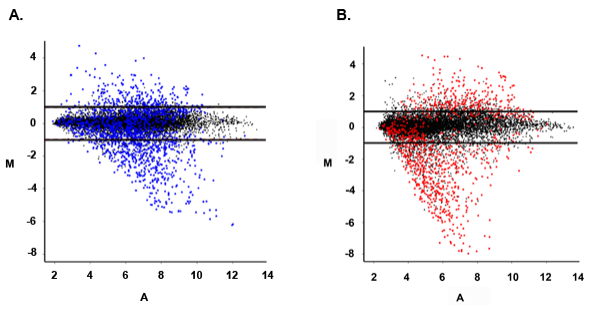
**Differential gene expression measurements made using IVT- and ribo-SPIA-based amplification methods. **Differential gene expression (M) and average intensity (A) were calculated by averaging results from replicate hybridizations performed using cRNA prepared by IVT (A) or sscDNA prepared by ribo-SPIA (B). Points outside the horizontal lines indicate probe sets with more than a 2-fold change in expression level for that sample preparation method. Blue points in (A) indicate probe sets with more than a 2-fold change as determined using *sscDNA *and red points in (B) indicate probe sets with more than a 2-fold change as determined using *cRNA*.

To test the ability to detect differential gene expression using amplified sscDNAs, we compared gene expression in two different RNA samples. We chose to compare K562 cell and sUHR RNAs since we have previously used these two RNAs to compare the performance of single round IVT-based amplification with other methods [25]. In Experiment 3, we did three separate sscDNA amplifications of K562 and sUHR RNAs. We used the same RNA preparations as for the previously reported IVT-based amplification experiments, but started with 1000-fold less material (10 ng instead of 10 μg). For each probe set, we used intensity values from replicate arrays to calculate relative gene expression (M, defined as log_2 _[mean K562 intensity/mean sUHR intensity]) and average signal intensity (A, defined as 1/2 log_2 _K562 mean intensity + 1/2 log_2 _mean sUHR intensity). M and A values obtained using sscDNA are shown in Fig. 3B and those obtained using IVT-generated cRNA are shown in Fig. 3A. The range of signal intensities was similar for the two methods, although the mean intensity was lower for sscDNA hybridizations (A = 5.22 for sscDNA, A = 6.12 for cRNA). In contrast, the range of M values was somewhat larger with sscDNA. In particular, the sscDNA method identified several transcripts that were more than 2^7^-fold higher in sUHR than K562 cell RNA (M < -7), but no differences of this magnitude were identified using cRNA. The number of probe sets associated with greater than 2-fold differences in expression (|M| > 1) was 1518 for sscDNA and 1043 for cRNA. 51% of the genes with >2-fold differences in expression on sscDNA arrays were not detected as >2-fold on cRNA arrays, but only 25% of the genes that were >2-fold different on cRNAs were not detected as >2-fold different on sscDNA arrays (Fig. 3).

**Figure 4:**
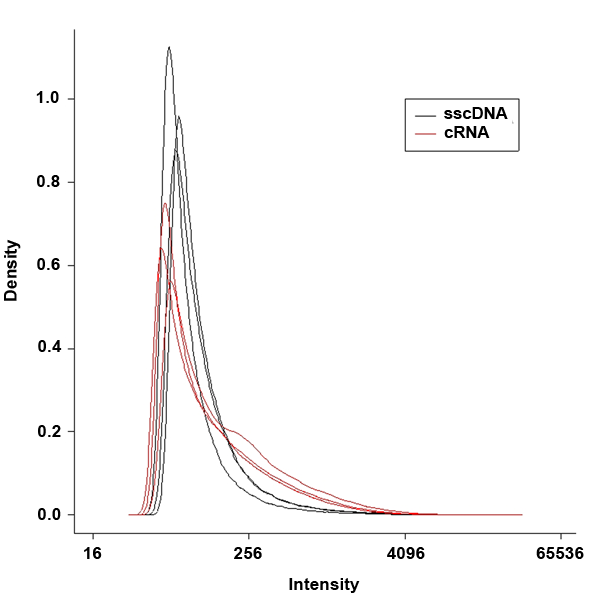
**Density plot of mismatch probe signal from cRNA and sscDNA targets. **The raw intensity distribution of all mismatch probes are plotted for three sUHR cRNA and three sUHR sscDNA arrays.

The new observation that sscDNA gave a wider range of relative expression (M) values despite lower average intensity (A) values could be explained by improved hybridization specificity under the conditions used in this study. This is plausible because the binding energy for DNA-DNA interactions is more sensitive to base pair mismatching than the binding energy for DNA-RNA interactions [26,27]. To look for further evidence about specificity of hybridization, we took advantage of the mismatched (MM) probes included on the arrays. For each perfect match (PM) GeneChip 25 mer probe, there is a corresponding MM probe with a single base mismatch at base 13. The MM probes were included in the probe set design to allow adjustments for nonspecific hybridization. Under ideal conditions, MM probes would never give signals higher than PM probes, although in practice this does sometimes occur. MM probes would be more likely to give stronger signals than PM probes if there was more non-specific hybridization of off-target sequences to the probes. We found that MM intensities exceeded PM intensities less frequently when we used sscDNA as compared to cRNA. When sUHR RNA was used as starting material, the average number of probe sets where MM intensity exceeded PM intensity was 2247 for cRNA versus 1671 for sscDNA (34% higher, p = 0.008). MM intensity also exceeded PM intensity more frequently with cRNA probes for K562 RNA arrays (2903 vs. 2482 probe sets, 17% higher, p = 0.017). When we looked at raw signal intensity for all MM probes, we found that the cRNA MM intensity distributions were skewed compared to the sscDNA MM distribution (Fig. 4). A closer examination of these distributions revealed that the use of sscDNA instead of cRNA resulted in a substantial reduction in the number of MM probes that gave relatively high intensity signals (Table 3). These findings strongly suggest that hybridization specificity is better for sscDNA than for cRNA. In a related study, Gingeras and coworkers [28] observed that increased nonspecific hybridization was observed when using directly labeled *E. coli *RNA as compared to cDNA. The increased nonspecificity was attributed to the presence of large amounts of rRNA in the samples. In our study however, both target preparations were prepared using oligo(dT) primers for the synthesis of first strand cDNA, so this explanation is less likely.

**Table 3:** Mismatch probe signal intensities from sscDNA and cRNA hybridizations.

**Target**	> 2× median*	> 4× median*	> 8× median*
sscDNA	14.1 ± 1.8%	5.0 ± 0.8%	2.0 ± 0.3%
cRNA	26.6 ± 1.4%	12.9 ± 0.9%	5.4 ± 0.6%

The proportion of mismatch probes with intensities greater than 2, 4, or 8 times the median for all mismatch probes on the same array. Values are mean ± standard deviation for triplicate arrays hybridized with sscDNA or cRNA prepared from sUHR RNA. Each array had 201,800 mismatch probes.

### Comparison of expression measurements made with sscDNA, cRNA, and qPCR

We wished to compare how measurements made using amplified sscDNA and microarrays compared with measurements made using other approaches. We began by comparing results obtained using sscDNA and cRNA microarray hybridizations for all 12,625 probe sets on the arrays. Since the sscDNA and cRNA methods would be expected to introduce different systematic biases, we were not surprised that direct correlations between signal intensities obtained with the two different methods showed show relatively poor agreement (r = 0.72–0.75 for K562 and r = 0.68–0.70 for sUHR, as opposed to r = 0.98–0.99 between replicates performed using the same sample preparation method). The finding indicates that it will not be useful to directly compare one array hybridized with sscDNA to another one hybridized with cRNA.

**Figure 5:**
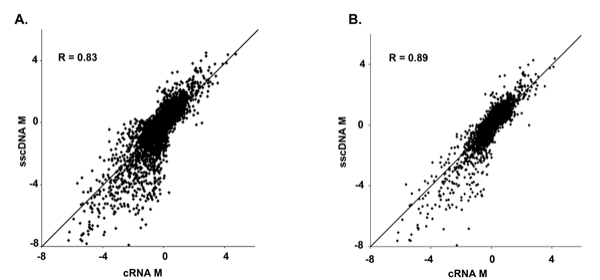
**Comparison of gene expression measurements between sample preparation methods. **(A) Differential gene expression measurements for all U95Av2 probe sets. (B) Differential gene expression measurements for 4179 probe sets after removal of signal less than the median intensity from both cRNA and sscDNA samples (A<5.485).

We next compared differential gene expression measures (M values) determined using sscDNA with those determined using cRNA. There was a clear correlation (r = 0.83, Fig. 5A). We expected that probe sets associated with low intensity signals would give less reliable measures of gene expression and when we removed these probe sets from the analysis the correlation improved (r = 0.90, Fig. 5B). On average, the estimated M values were slightly larger (~1.2 times higher) when sscDNA was used instead of cRNA.

Next we generated another set of expression measurements that could be used as a basis for comparison for the sscDNA and cRNA array results. qPCR is typically used as "gold standard" to confirm putative differentially expressed genes detected with microarrays. Since we saw a subset of genes for which expression differed between sscDNA and cRNA targets, we next assessed if either method tracked more closely to qPCR. We chose qPCR primers and probe sets from a large group of >1000 sets that have been developed for various studies. From these, primers and probes for four subsets of genes were selected for qPCR. The first set included all genes with >4 fold difference in expression between K562 and sUHR samples as determined using the sscDNA method, the cRNA method, or both methods (53 primer/probe sets). The second set included all other genes in which the two methods disagreed by more than 2-fold (29 primer probe sets). The third set consisted of a group of 33 empirically-derived 'housekeeping genes.' These were all genes that were nearly equally expressed (|M| < 0.1) in K562 and sUHR samples according to both the sscDNA and cRNA methods and gave strong signals (A > 5 for both methods). The fourth set included 8 housekeeping genes that had been previously validated as controls for qPCR in other experiments. We determined the gene copy number for each qPCR primer and probe set and then calculated a measure of relative expression, M = log_2 _(K562 copy number)/(sUHR copy number), that could be directly compared to M values from arrays. 37 putative duplicate probe sets from 17 genes probe sets were hand-curated to confirm that they would correspond to the predicted qPCR product. In two cases probe sets were found to be misidentified in the GeneChip annotation and were removed from the analysis. In the remaining cases of duplication, the qPCR and microarray values were averaged across the duplicates. The final set of 106 curated genes and the associated data can be found at .

**Figure 6:**
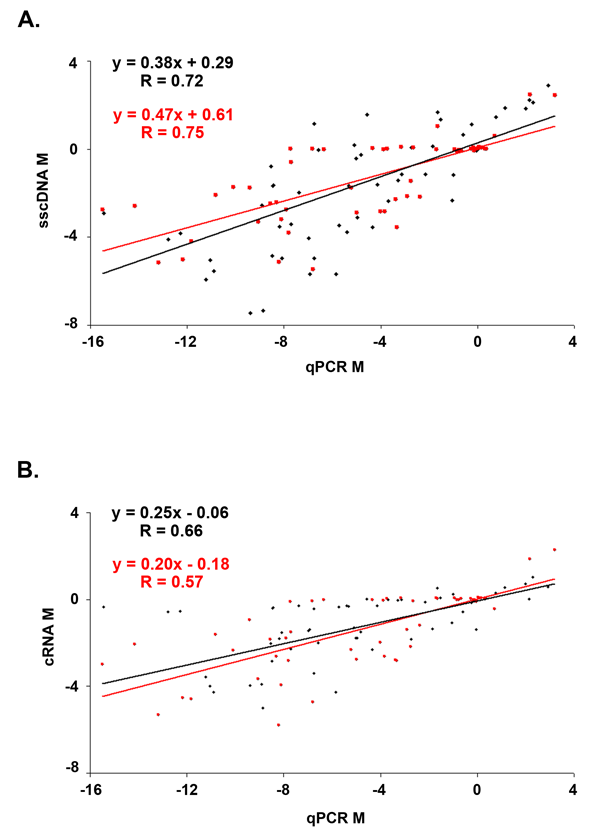
**Comparison of differential gene expression measurements between qRT-PCR and microarrays. **Differential gene expression measurements for 106 genes made using qRT-PCR were compared to array measurements made using sscDNA targets (A) or cRNA targets (B). Red points indicate genes for which cRNA and sscDNA samples varied by more than 2-fold in differential gene expression.

There were clear correlations between qPCR M values and array M values obtained using sscDNA (Fig. 6A) or cRNA (Fig. 6B). When all 106 genes were included, qPCR results correlated slightly better with sscDNA (r = 0.72) than with cRNA (r = 0.66). When we included only the 29 genes for which sscDNA and cRNA methods disagreed by more than 2-fold, the difference between the two sample preparation methods became more pronounced (r = 0.75 for sscDNA vs. r = 0.57 for cRNA). Not surprisingly, both array-based methods tended to give smaller estimates of M than qPCR; this relative underestimation was somewhat less marked for the sscDNA than the cRNA method. In summary, results from arrays hybridized with sscDNA samples amplified using the ribo-SPIA method tracked with qRT-PCR more closely than did results from arrays hybridized with cRNA samples prepared using the traditional IVT method.

## Conclusion

We examined the suitability of a new isothermal linear amplification method for application to Affymetrix GeneChip microarrays. We performed a series of tests using starting amounts of RNA ranging from 5 to 100 ng for amplification yield and reproducibility. The amplification reactions consistently produced sufficient sscDNA for multiple array hybridizations. Pairwise comparison of technical replicates hybridized to microarrays by regression analysis showed excellent consistency. When we used sscDNA to analyze differential gene expression between two samples, we found a larger dynamic range than that obtained with cRNA hybridizations. The improved performance appears to be related to increased sscDNA hybridization specificity. The data obtained using this new method also more closely matched the results from qRT-PCR than data obtained using standard IVT reactions, even though the amount of starting RNA used was 1000-fold less. This new amplification method is a useful alternative approach for preparing targets that is especially well-suited for experiments involving small amounts of starting material.

## Methods

### Test samples

Clontech Human Universal Reference Pool total RNA (cUHR), derived by pooling RNA from a variety of human tissues, was purchased from BD Biosciences and used in Experiment 1. Mouse total liver RNA was isolated by standard methods from C57/BL6 mice according to procedures approved by the UCSF Committee on Animal Research and used in Experiment 2. For Experiment 3, we used Stratagene Human Universal Reference Pool and K562 erythroleukemia total RNAs from the same batches used in a previous study [25]. All samples were assessed for size and integrity using the Agilent 2100 BioAnalyzer RNA 6000 Nano LabChip assay. RNA and DNA samples were quantified using a NanoDrop ND-1000 spectrophotometer.

### Sample preparation

sscDNA samples were prepared using the NuGEN Technologies Ovation RNA amplification and Biotin Labeling system (Version 1.0) according to the manufacturer's directions from the indicated amount of starting RNA (5–100 ng). All reactions were performed in 0.2 ml strip PCR tubes in an MJ GeneWorks PTC-100 thermocycler using recommended programs. Since the seal for PCR tubes and caps tends to deteriorate with repeated use, we replaced the caps for each tube before each resealing step in the protocol. Following amplification, sscDNA product was purified using QIAquick PCR purification kits (Qiagen). Samples were fragmented and end labeled with biotin. After stopping, each reaction was concentrated in a Microcon YM-3 column to a final volume of ~20 μl. The concentrated material was purified using a Centri-Sep 100 spin column (Princeton Separations). Negative control reactions were prepared by replacing input RNA with the appropriate volume of RNase free water.

### DNA microarrays

All samples were placed in standard Affymetrix hybridization buffer. The sample denaturation time for the sscDNA samples was reduced from 5 to 2 minutes and hybridization time increased from 16 to 20 hours as recommended by NuGEN Technologies. cUHR gene expression was assayed using Affymetrix Human Genome U133A GeneChip arrays (Experiment 1). Mouse liver RNA was assayed using Murine Genome Mu6500A arrays (Experiment 2). K562 and sUHR RNAs were assayed using Human Genome U95Av2 arrays (Experiment 3). One template independent sample was also analyzed using a Human Genome U95Av2 array (Experiment 4). Arrays were stained with phycoerythrin-streptavidin according to the manufacturer's instructions. Metrics for all sample hybridizations including scaling factors, mean background intensities, and percent present calls have been provided (see Additional File 1). Each set of data was normalized independently using RMAExpress software . K562 and sUHR microarray data were also analyzed using Microarray Suite 4.0 in order to calculate PM-MM values for each transcript probe set. Probe level analyses were performed using the BioConductor [29] affy analysis package [30]. All microarray data have been deposited in the Gene Expression Omnibus (GEO) database under the accession numbers GSM41384 – GSM41393, GSM41433 – GSM41438 and GSM4843 – GSM4847.

### Real-time PCR

Real-time (RT) PCR was used to measure the expression of selected genes in sUHR and K562 cells. Gene-specific primers for multiplex real time RT-PCR were designed for each gene of interest using "Primer Express" software (Perkin-Elmer) and purchased from Biosearch Technologies. Sequence data for all oligonucleotides primers has been provided (see Additional File 2). First strand cDNA synthesis was performed using total RNA, Powerscript reverse transcriptase (BD Biosciences), and random hexamer primers. Real time amplification was performed using an ABI Prizm7900 and Invitrogen Universal Master Mix. Relative gene copy numbers (GCN) were calculated as described previously [31]. GeNorm [32] was used to select the two most stable housekeeping genes across all specimens for normalization.

## Authors' contributions

CSB conceived of the study, participated in the design and coordination, and wrote the manuscript. CG participated in the design of the study and both CG and KH performed the microarray experiments. GMD carried out the real time PCR studies. JYHY performed the statistical analyses. DJE participated in the study design and analysis and revised the manuscript. All authors read and approved the final manuscript.

## Supplementary Material

Additional File 1 Hybridization Metrics is a .txt file suitable for opening in Microsoft Excel containing information about each individual hybridization. Click here for file

Additional File 2 Taqman Primers provides sequence information for oligonucleotides used for qRT-PCR. Click here for file
